# “We have a lot of battles to fight”: The experiences of older, Black, same-gender-loving women during the COVID-19 pandemic

**DOI:** 10.1016/j.ssaho.2025.101648

**Published:** 2025-06-27

**Authors:** Robynn Ashenden, Porsha Hall, Mary Anne Adams, Dipa Sharma Gautam, Jennifer Horn, Tonia Poteat

**Affiliations:** a Department of Health Behavior, Gillings School of Public Health, University of North Carolina at Chapel Hill, 170 Rosenau Hall, 135 Dauer Drive, Chapel Hill, NC, 27599, USA; b ZAMI NOBLA: National Organization of Black Lesbians on Aging, P.O. Box 90986, Atlanta, GA, 30364, USA; c Department of Social Medicine, University of North Carolina School of Medicine, 333 S Columbia Street, Chapel Hill, NC, 27599, USA; d Pikes Peak Area Agency on Aging, 14 S Chestnut Street, Colorado Springs, CO, 80905, USA

**Keywords:** COVID-19, Health equity, Social determinants of health, Sexual minority, African American, Intersectionality

## Abstract

Previous studies show mental and physical health inequities have been exacerbated by the COVID-19 pandemic, but few use an intersectional lens to identify the assets and unmet needs of groups that are multiply marginalized by their social categories. This study explores how older, Black, same-gender-loving women discussed their experiences during the COVID-19 pandemic with a focus on health. Sixteen focus groups were conducted between July and December 2020 with 4–8 participants each (N = 102) from across the U.S. Content analysis revealed insight into the determinants of health among older, Black, same-gender-loving women, impacts of the pandemic, and how participants were able to maintain their health. Participants’ experiences with healthcare were most prominently shaped by their intersectional social categories, as they faced complex systems of oppression rooted in heterosexism, racism, sexism, and ageism. We found discrimination to be the most prominent determinant of health, manifesting through various barriers and a lack of access to providers of preferred social categories. The complex trauma experienced during the Trump Administration, COVID-19 lockdowns, and racial unrest after the murder of George Floyd had generally negative impacts on daily life, relationships, and mental health. However, these were mitigated for some through positive internal and external coping strategies, including online resource use, mutual aid, and telehealth experiences. As evidenced by these experiences, future research and interventions need to adopt an intersectional lens and center multiply marginalized groups to mitigate future harms and promote health equity.

## 1. Introduction

The exact number of older, Black, same-gender-loving (i.e., those who are attracted to people of the same gender, and therefore may also identify as lesbian, gay, bisexual, or queer) women in the United States is unknown. However, an estimated 1.2 million Black adults in the U.S. self-identify as lesbian, gay, bisexual, or transgender (LGBT), where 60.9 percent also identify as women and 20 percent are over the age of 50 (Choi et al., 2021). When compared to their White counterparts, Black LGBT older adults report higher lifetime levels of LGBT-related discrimination, social isolation, and stigma, and lower household income, educational attainment, and social support (Kim, Jen, & Fredriksen-Goldsen, 2017). These chronic experiences with multiple forms of discrimination, stigma, and structural oppression produce inequities across multiple measures of physical and mental wellbeing (Movement Advancement Project, 2017).

Discrimination acts as a social determinant of health both directly (i. e., by increasing chronic stress) and indirectly (i.e., by shaping other determinants such as income). These inequities are compounded by the fact that older Black lesbian, gay, bisexual, transgender, or queer (LGBTQ) adults are also more likely to go untreated by healthcare professionals (Kum, 2017). They experience a range of barriers, including a lack of high-quality healthcare centers in proximity to homes (de Mendonça Lima & Ivbijaro, 2013), lack of health insurance (Rostain et al., 2015), lack of transportation (Rostain et al., 2015), fear of discrimination (Wylie et al., 2016), and prevalence of stigma against accessing mental health care (McDermott et al., 2018).

Intersectionality, as theorized by Black feminist thinkers and activists, can be used to conceptualize how older, Black, same-gender-loving women are multiply marginalized through experiences with ageism, racism, sexism, and sexual orientation discrimination (Bowleg, 2008; Crenshaw, 1989, 1991; Kum, 2017). The intersectionality framework proposes individuals have overlapping axes of social categories (race, age, sexual orientation, etc.), with structures of power creating inter-locking systems of oppression and/or strengths along these axes (Crenshaw, 1991). Each axis cannot be viewed in isolation, i.e., Black women do not face misogyny and racism additively (Crenshaw, 1989). Rather, they combine to form multiaxial matrices that produce unique experiences with discrimination, such as through misogynoir or the intersection of racism and sexism as experienced by Black women (Bailey, 2021; Collins, 2000; Farmer, 1996). These axes also produce intersectional strengths within community, through different assets available depending on one’s intersecting social categories (Mbah et al., 2022).

Existing research reveals mental and physical health inequities experienced by older adult (Hausner et al., 2021), Black (Vasquez Reyes, 2020), and LGBTQ (Nowaskie & Roesler, 2022) communities have been exacerbated by the COVID-19 pandemic (Dahlberg, 2021). However, research specifically on the impact of the pandemic on older, Black, same-gender-loving women is scant. While this paper only explicitly discusses the experiences of older, Black, same-gender-loving women during the early phases of the pandemic, many of the issues raised pre-dated and continue to be exacerbated by the pandemic today. Studying these early stages provides insights into where support systems and interventions failed vulnerable populations in times of emergency, informing strategies for equitable recovery, resilience-building, and more effective interventions and preparation for future crises. Additionally, the majority of research regarding health inequities during the pandemic has used a deficits lens towards multiply marginalized communities. The lack of assets-based approaches to health promotion has resulted in a reduced understanding of the strengths and coping abilities of these communities during the pandemic. More research into the lived experiences of older, Black, same-gender-loving women is needed to fill these research gaps and address health inequities (Adams & Poteat, 2016). Through qualitative methods, this study investigates the experiences of older, Black, same-gender-loving women during the early stages of the pandemic, with the goal of identifying both current assets and needs for future interventions and community programs.

## 2. Methods

We conducted focus group discussions (FGDs) with Black, same-gender-loving women aged 40 years and older. Led by co-Principal Investigators from each organization, this community-engaged participatory research study represented a partnership between the University of North Carolina School of Medicine and ZAMI NOBLA: National Organization of Black Lesbians on Aging. Through this partnership, this exploratory study was initiated to examine the impacts of the pandemic and to identify the assets and unmet needs within the constituency of ZAMI NOBLA. Consistent with methods outlined by Kim, Sefcik, and Bradway (2017), this study used a Qualitative Description (QD) approach, which describes research that focuses on gaining insights about events and/or experiences from informants to later develop interventions, specifically in healthcare and nursing-related research. FGDs were selected, as they have been shown to be a culturally responsive method to facilitate empowerment through knowledge-exploration and community capacity-building (Hall et al., 2022). Criterion sampling (Moser & Korstjens, 2018) was used to select participants. The criteria of importance were residence in the U.S., age of 40 years or older, self-identity of Black or African American, female sex assigned at birth, and self-identity of lesbian, gay, same-gender-loving, bisexual, pansexual, or queer. The recruitment methods for participants included electronic flyers, shared via Facebook and listservs, and word-of-mouth via existing participants recruiting others in their social networks who also met eligibility requirements.

Between July and December of 2020, sixteen FGDs were conducted via HIPAA-compliant Zoom. Each FGD consisted of 4–8 participants (N = 102), one facilitator, and 1–2 notetakers. FGDs were organized using a semi-structured guide, lasting approximately 90-minutes each. The semi-structured focus group guides contained questions pertaining to the pandemic’s effect on participants’ physical health, mental health, nutrition habits, economic status, and relationships with friends/family. In addition, semi-structured guides included general questions about participant experiences during the pandemic with access to testing and general care, experiences with telehealth, coping strategies, mutual aid, preferred healthcare provider identity, and willingness to get vaccinated. After the FGD, participants completed an online demographic survey (Table 1) and were remunerated $50. Throughout the data collection the study team met biweekly for data validation purposes.

### 2.1. Reflexivity

The FGD facilitator and notetakers all identify as cisgender, Black lesbians. All co-authors identify as cisgender women, with four also identifying as Black and five as sexual minority women. Study team members considered their own experiences and identities during analysis and debriefing to distinguish participant findings from their own perspectives.

### 2.2. Ethics

Ethical approval was provided by the Institutional Review Board at The University of North Carolina School of Medicine. To promote confidentiality, no individual identifiers were retained after remuneration, and no written consent forms were used. Rather, consent for participation was collected verbally after the oral consent form was read to all individuals and ample time and opportunity to ask questions was provided. The funder of the study did not play any role in the collection, analysis, or presentation of the data.

### 2.3. Analysis

A manifest analysis of content was conducted with a directed approach in line with Bengtsson (2016) and Hsieh and Shannon (2005). FGDs were audio-recorded and transcribed verbatim by a professional transcription company. Each transcript was reviewed by an analyst for accuracy prior to coding. The codebook contained *a priori* codes and was expanded during this review process. Analysts met with the larger study team (including the FGD facilitator and one notetaker) to discuss this expansion. Once the codebook was finalized, two independent analysts coded the sixteen transcripts in ATLAS.ti, with double-coding two. Inter-coder reliability was 0.871, assessed using Krippendorff’s c-α-binary. Themes were identified (via exploration across and within codes) and organized in written summary matrices and memos. Regular meetings with the data analysts, ZAMI NOBLA organization members, and other community leaders in LGBTQ health and/or aging served as a way to validate and triangulate data interpretation, informing the study results below.

## 3. Results

As presented in Table 1, the mean age of participants was 57. 46 % of participants were single, and 26 % were married. 43 % of participants lived with partners and 21 % with children. Of note, 41 % of participants held a master’s degree.

A total of three themes were identified (Table 2). Four concepts offered insight into the first theme of determinants of health among older, Black, same-gender-loving women both during the pandemic and outside of it. Four concepts contributed to the second theme, elucidating impacts of the pandemic. Three concepts discussed how participants maintained their wellbeing during the pandemic, making up the third theme.

### 3.1. Theme 1: Determinants of Health

#### 3.1.1. Concept 1: Intersectional Social Categories

The most common social categories recognized by participants as shaping experiences were race, gender, age, and sexuality. They noted the combinations of their social categories produced unique experiences that would not arise additively, reflecting an emergent view of intersectionality.

When we’re talking about Black lesbians, at least I know for me, it’s not even that being a lesbian is my primary identity, because my experience of being a lesbian is shaped by my Black identity … and when we’re talking about queer folks who are older, that doesn’t really exist.

(Participant 71, FGD10).

Participants indicated social categories determined experiences with healthcare by acting as barriers in different ways: *“sometimes you wonder if you’re being dismissed because you’re Black or because you’re gay or both”* (Participant 72, FGD10). These experiences were also shaped by how visible certain social categories are in the healthcare system, stating race was often a driving factor despite their other social categorizations: “if you’re lesbian, we have a lot of battles to fight and we got to try to keep it on the down low. But you can’t do color like that. You can’t do that” (Participant 110, FGD15).

Intersectional social categories also influenced participants’ experiences and behaviors during the pandemic. Participants stated their communities were treated as *“expendable”* (Participant 90, FGD13); they were both more affected and received fewer resources. Participants thus saw their race and age as risk factors due to the disproportionate impacts seen among older Black people. Subsequently, they stated they took more precautions than peers in social distancing and personal protective equipment (PPE).

Identifying as Black, the political climate in 2020 also affected participants’ experiences during the pandemic. Numerous participants mentioned finding difficulty in separating the impacts of the Trump Administration, the murder of George Floyd and following unrest, and COVID-19: “It’s been hard in some cases to extricate COVID from Floyd, 45 … there is politics in life, period. And COVID is another vehicle with which those things get played out” (Participant 116, FGD16).

#### 3.1.2. Concept 2: Systematic Barriers

The most noted barrier to healthcare was discrimination. Participants believed non-Black providers were not aware of the nature of the *“Black body”* (Participant 11, FGD2) and what care is appropriate for them. Many participants indicated being stereotyped by White physicians based on their weight and/or due to being a Black woman: “[White doctors] have certain assumptions about what a large Black woman has healthcare wise. They have this imaginary list” (Participant 26, FGD4). Numerous participants also expressed being *“denied proper care”* (Participant 11, FGD2) due to being Black women.

I went to my doctor, and there’s a male, this White guy. And I asked him to, you know, I told him I wanted to have my tubes tied. And he looked at me real wicked like, out the side of his eye. And he told me no.

(Participant 110, FGD15).

Discrimination also manifested as dismissals of pain of Black individuals under a White-dominated system, as well as a systemic lack of healthcare resources due to racism towards communities of color: “if you want to go to a good hospital, get out of the community of color and go to another community. If you want a good doctor, a good hospital, you will not find it in your community” (Participant 111, FGD15).

These experiences with discrimination in the healthcare system compounded the inequities during the pandemic.

[The inequities] don’t surprise me because of the treatment that I personally have gotten in the health system, where I feel like my voice isn’t heard, where I feel like my concerns aren’t addressed … I’ve encountered that so many times that it doesn’t surprise me that a person with COVID who was Black or Brown might have a harder time getting someone to take them seriously, not tell them that, ‘Oh, it’s just a cold, it’s just whatever, don’t worry about it. Come back later when you’re almost dead’.

(Participant 20, FGD3).

However, participants made the distinction that the pandemic was not the cause, but only brought existing systematic barriers to light:

You’ve been treating us like this long before the movement, long before you put your foot on and killed George Floyd. You knew what the issues were. And so, they know what the issues are in terms of healthcare. We don’t trust the healthcare system.

(Participant 111, FGD15).

#### 3.1.3. Concept 3: Provider Preferences

When looking for a specialist or dentist, participants tended to prioritize skill. However, when looking for a primary care, mental health, or OB/GYN provider, social category was most important. Gender was mentioned most often by participants as being one of, if not the, most important requirement when choosing a provider. Participants preferred women because “a female would be more likely to understand what is going on with [my] body than a male” (Participant 39, FGD5). They also believed female providers were more sensitive and understanding towards mental health and sexuality. Race was also important, as participants mentioned Black doctors make them feel more comfortable and provide them appropriate care. Additionally, Participants did not want the burden of explaining or teaching providers about LGBTQ-specific issues, and thus were more comfortable with providers who are themselves part of the LGBTQ community. Due to the difficulty of finding LGBTQ providers, participants stated if providers were at least LGBTQ-affirming they would still be able to “*sympathize*” (Participant 9, FGD2).

While there was consistency across race, gender, and sexuality, there was variability in preferred age of providers. Some participants mentioned they prefer young doctors because they felt them to be more competent culturally and in medical knowledge. Some noted younger providers spend more time with patients, taking copious notes and communicating well with patients. On the other hand, others preferred older providers because they are more experienced. Even further, some participants preferred middle aged providers because “if they’re too young, they don’t have enough experience, and if they’re too old, they may not be keeping up on the latest information and medical stuff” (Participant 48, FGD6).

#### 3.1.4. Concept 4: Medical and Governmental Distrust

Historical and current experiences with the healthcare system, pharmaceutical companies, and the government produced feelings of uncertainty and distrust among participants. This was especially influential in participants’ decisions about receiving the COVID-19 vaccine; most wanted to ‘wait-and-see’ others receive the vaccine. The next largest group were not interested in the vaccine at all, with only 10 remaining participants immediately willing to receive the vaccine. Among those who wanted to ‘wait-and-see’, participants generally stated one of three prerequisites for feeling safe to take the vaccine. Most commonly, participants required more testing and information about the trials, efficacy of the vaccine, and side effects/interactions, as many doubted the vaccine would work in their body: “Vaccines are developed with normal weight, White people in mind, and I’m a fat Black person. And so would the vaccine actually even be effective in my body?” (Participant 10, FGD2).

The second requirement was seeing the effects of the vaccine on White people. The third requirement was seeing certain public figures endorse the vaccine, including former president Barack Obama and scientific figures: *“I would take it if Dr. Fauci said so”* (Participant 64, FGD9).

Participants described various reasons for COVID-19 vaccine distrust, including general vaccine mistrust and distrust of the Trump administration’s role in vaccine development.

[I am a] serious believer in every other vaccine I’ve gotten. I’ve got shingles vaccine, flu, pneumonia, all that kind of stuff. I love it. I think vaccines are great. But the stuff that’s coming out of, you know, Trump with this whole confusion, this craziness he’s been generating. I wouldn’t.

(Participant 60, FGD8)

If it happened under the current administration? Absolutely not, because I don’t trust them. And I don’t trust who they trust. If it was possible to go to another country and have somebody else do it, I would do it in another country.

(Participant 47, FGD6).

Others reported distrust towards pharmaceutical companies, believing they only care about money and not public health or safety of vaccines.

### 3.2. Theme 2: Impacts of the Pandemic

#### 3.2.1. Concept 5: Health Impacts

Participants noted impacts of the pandemic on their mental health more often than their physical health. Mental health impacts were generally negative, due to increased stress and unpredictability. The most common causes of stress was fear of contracting COVID-19. Moreover, being aware of health inequities during the pandemic impacted mental health: “it’s horrible for our mental health in terms of knowing the disproportionate impacts COVID-19 is having on us” (Participant 15, FGD3). Many participants also noted that social isolation had a negative effect on their mental health.

The amalgamation of political events in 2020 exacerbated the mental health impacts of the pandemic by amplifying feelings of anxiety and isolation:

I think if you are an individual who feels being a lesbian is an isolating experience, being an African American in this political climate has made you more inclined to isolation. You are more of those things now than you’d have been when we had more options [before the pandemic].

(Participant 115, FGD16).

Participants also mentioned the physical impacts of the pandemic, which were also generally negative. Participants were doing less physical activity due to pandemic-related restrictions, with one noting they “*started getting cramps”* from inactivity and gained weight (Participant 3, FGD1). Overlap between physical and mental impacts was observed, with the closure of amenities impacting not only their physical health but also intensifying feelings of isolation.

#### 3.2.2. Concept 6: Impacts on Daily Life

Outside of health, the most discussed impact of the pandemic was changes to daily life, including eating habits, hygiene habits, and travel. The pandemic lead to overeating and snacking as a way of coping; participants stated *“it’s hard not to graze*” being home more often (Participant 4, FGD1). Additionally, a few of the participants mentioned they were fearful of buying fresh produce during the pandemic:

I wouldn’t be able to clean it enough to assure that if somebody else had walked by the produce section and had COVID that I wouldn’t catch it from that. So, maybe that’s overthinking it, but I was scared to buy the fresh fruits and vegetables.

(Participant 20, FGD3).

Participants also stated their hygiene habits were negatively impacted by isolation. For example, a participant noted since she was not seeing anyone in-person, she was not taking showers as regularly and often not changing out of pajamas. Participants’ daily lives were also affected as they avoided travel.

#### 3.2.3. Concept 7: Relationship Impacts

The pandemic had salient impacts on participants’ relationships with partners, dating, family, and friends. Participants who were already in intimate relationships got to spend more time together, which had varying effects. For some it had positive impacts: “it’s just been wonderful in terms of giving us time to work through stuff, time to enjoy each other’s company, to you know, just … It’s made us closer” (Participant 60, FGD8). However, other participants mentioned unique challenges due to living together during the pandemic. As one explained, “it’s been challenging because I’m a rabbit, when I feel offended or feel like I need to be on guard, I’ll run, I’ll escape, I’ll go to a hotel or whatever. And that’s really not an option” (Participant 52, FGD7). This caused stress and strain in relationships: “I’m home more and with my girlfriend more. And I think being around her more, and her family, has just caused much more mental stress than I was prepared for” (Participant 85, FGD12). A few participants also mentioned parental caregiving roles during the pandemic impacted their intimate relationships. They avoided meeting their partner to keep their parents safe from the virus, and when they met, they took precautions to minimize the risk of contracting the virus.

Those attempting to date found it harder during the pandemic, as it was more difficult to make meaningful connections not in-person. However, some participants found *“creative ways”* of dating by using online resources: *“we’ve had dining together over Skype”* (Participant 54, FGD8).

The pandemic also impacted the participants’ ability to socialize with friends and family. They expressed that keeping distance was better than putting their own and their loved ones’ health in risk. However, not seeing friends and family was hard: “I have kids and grandkids. We’ve decided that this year is a wash. They’re not gonna come. And that hurts” (Participant 60, FGD8).

#### 3.2.4. Concept 8: Positive Impacts

Despite more negative impacts being discussed, there were some instances of positive impacts of the pandemic. Working from home gave participants more time and freedom to rest, sleep, be creative, and perform self-care. The pandemic also gave participants time to reflect and appreciate what they had: “I think I got some inner peace, and I took time to listen to myself and realize that I think I’m living in the good ages. And I’m happy” (Participant 45, FGD6).

While the pandemic did increase isolation for most, some found new connections through online platforms. It even made connecting with family easier for some, as participants were able to communicate with people via Zoom whom they wouldn’t be able to see in person:

In some weird kind of way, I can see some of the silver linings connecting with family via Zoom that I haven’t seen in 40 years. That would not have happened [if not for the pandemic], I don’t think. Going inside to figure out how to keep connections with people.

(Participant 116, FGD16).

### 3.3. Theme 3: Maintenance During the Pandemic

#### 3.3.1. Concept 9: Coping

The majority of coping strategies used by participants were described as positive. For some participants, these were completely new strategies, but for others they were building on existing strategies where they now had to *“practice them more frequently”* (Participant 90, FGD12). Positive coping strategies usually related to ameliorating the mental health impacts of the pandemic via independent self-care skills. These included, but were not limited to, engaging in journaling, painting, sewing/crocheting, reading, writing, puzzles, meditation, sound healing, and coloring with friends: “we spend 2 hours, we pick out a color, we pick out a picture, we color, and we show it to each other. And that is bringing me so much joy right now” (Participant 62, FGD9). Participants also used physical activity outdoors to relieve stress, such as hiking and electric biking: “I need to be able to sweat. That really decreases the stress that I get” (Participant 21, FGD3). Other positive coping strategies centered around enhancing social interaction and support. For participants who lived alone, sending letters/calling loved ones and engaging with their church helped combat the negative effects of isolation.

Many participants indicated utilizing online resources to maintain both their physical and mental health during the pandemic, generally having positive experiences:

One of the things that it’s helped me cope is Zoom. Being able to like tap into the resources of having online conversations, online classes, just being able to see who I’m talking to because on the phone, it’s just not enough. And I’m like, this is some kind of substitute, the internet having these kinds of conversations is some kind of substitute. So, that’s helpful to cope.

(Participant 20, FGD3)

The most common activity in which online resources were used was yoga, primarily through classes on Zoom. Other forms of physical activity conducted virtually included meditating, kickboxing, and Tai Chi over Zoom and/or YouTube. Some participants also used online resources as a creative outlet, finding inspiration on Pinterest or attending virtual concerts. Participants also used online resources to engage in social interaction and cope with isolation. Zoom and FaceTime were used most often to communicate with friends and family. Zoom, Skype, and Facebook were also used by participants to communicate with partners or date. Zoom was also used to maintain social connections to larger communities, with multiple participants connecting with spiritual centers and other community groups.

Albeit less commonly discussed, all negative coping strategies mentioned related to not taking care of one’s physical health. The most common negative coping strategy was overeating and snacking.

#### 3.3.2. Concept 10: Support

Mutual aid was the most common type of support received and provided by participants. Many participants were involved in food related mutual aid organizations, both as receivers—“yesterday I called, and they came and actually delivered it to my home yesterday, and then put me on the list for continued support *of the food service”* (Participant 13, FGD2)—and providers—“a local church, one that’s not far from me, every Wednesday they advertised picking up boxes of food, and this food consists of potatoes and onions and fruit and that type of thing. I was able to pick up a couple boxes for seniors in my area” (Participant 12, FGD2). The second most discussed type of support was economic support via governmental aid, through social security and stimulus checks.

#### 3.3.3. Concept 11: Telehealth

Most participants engaged in telehealth during the pandemic at some point, generally for mental health services. Some participants sought new mental health services virtually because of the pandemic, and others transitioned from in-person to telehealth. In most cases telehealth was a new experience for the participant.

Most telehealth experiences were characterized as positive and some even preferred telehealth. Some benefits of telehealth were the enhanced personal experience and feeling less rushed: “It’s longer and it’s more relaxed … So, I think I’m liking it more this way than I was the other way” (Participant 25, FGD4). Logistical barriers were eliminated by telehealth, such as having to schedule time off work or incorporate travel time: “[Telehealth] helps me because I don’t have to catch two buses to go to my primary care physician, or my therapist” (Participant 88, FGD12).

While negative telehealth experiences were not as commonly discussed, when they were raised, they were most often in the context of physical health. Participants indicated they did not think they were receiving physical healthcare of the same quality over telehealth:

I send the pictures when I have my flare ups, but I don’t think they can see them … They keep thinking I have hives, and I’m saying no, they’re not hives … So, when it comes to my lupus diagnosis, I’d rather go in, especially if I’m having a flare up, because I don’t think I’m getting a just medical assessment with the telemedicine.

(Participant 41, FGD6).

## 4. Discussion

This study fills an important gap in the literature on health determinants and impacts during the early phases of the COVID-19 pandemic as it provides a unique perspective from a population often neglected by health research: older, Black, same-gender-loving women. The themes identified in this study are both consistent with and distinct from previous literature. Social categories of participants were the most crucial determinants of health, shaping all experiences with healthcare. In line with Bowleg (2008), participants viewed social categories as intersectional, producing unique experiences what would not exist additively between their social categories. Members within multiply marginalized social categories face magnified forms of discrimination on structural, interpersonal, and individual levels, having cumulative impacts on health outcomes. Consistent with this, participants indicated facing complex structural barriers to care including heterosexism, racism, sexism, and systemic lack of access to resources (Simpson & Helfrich, 2014). These experiences contribute to the prevalence of medical distrust and lack of interest in vaccine uptake expressed among participants (Casanova-Perez et al., 2022, pp. 275–284; Woody, 2013).

Positive interpersonal patient-provider experiences were seen to mitigate the harms caused by discrimination in healthcare. When providers hold social categories similar to patients, or are at least knowledgeable about the experiences of other social categories, patient-provider interactions are more likely to be positive and thus promote trust (Rahman et al., 2023). This was particularly relevant with relation to gender, race, and sexual orientation; however, many participants noted difficulty in accessing providers of preferred social categories. This study thus adds to existing literature by suggesting lack of access to preferred providers is a barrier experienced by aging, Black, same-gender-loving women (Seelman et al., 2017). Emphasizing cultural humility trainings and increasing the presence of LGBTQ women of color in healthcare settings may be an avenue to combat reproduction of harms on the multiply marginalized.

Structural and individual experiences with multiple axes of discrimination led to profound impacts during the pandemic for older, Black, same-gender-loving women. Communities of color have been disproportionately impacted by the COVID-19 pandemic, producing negative impacts on mental health outcomes due to increased exposure, cumulative burden, and isolation (Saltzman et al., 2021). In support of previous literature, participants indicated experiencing disproportionate impacts due to complex trauma; not only navigating the pandemic, but also structural racism and the political climate under the Trump Administration (Novacek et al., 2020; Ibrahimi et al., 2020). While various factors affected participants’ mental health, their experiences with systemic racism were most salient; participants viewed the impacts of COVID not singularly, but as one component in a larger system of racism and oppression.

The decline of mental health outcomes during the pandemic has been associated with an increase of physical inactivity and unhealthy diets, especially amongst the aging population (Mattioli et al., 2020; Robinson et al., 2021; Woods et al., 2020). Participants in this study corroborate these findings, reporting less physical activity due to the closure of recreational facilities, less fruit and vegetable consumption, and more snacking, contributing to weight gain during the pandemic. Physical inactivity and poor diets have long term negative effects on health, including decreased ability of the body to fight infections, increased risk of diabetes-related harms, and increased risk of diseases of the immune, respiratory, cardiovascular, and musculoskeletal systems (Fisher et al., 2020; Mattioli et al., 2020; Woods et al., 2020). Since older, Black, same-gender-loving women are at a higher risk for these conditions due to natural processes of aging and prolonged experiences of discrimination and chronic stress (Din-Dzietham et al., 2004; Meyer, 2003), it is crucial to promote physical activity and access to healthy meals during periods of isolation especially among this population.

Declining mental health during the pandemic has also been associated with COVID-19 impacts on intimate relationships (Rodriguez-Dominguez et al., 2022; Ahuja & Khurana, 2021). Compared to the pre-lockdown period, relationship satisfaction and perceived quality of relationships has decreased, correlating to increased partner conflict and other difficulties cohabitating or communicating (Rodriguez-Dominguez et al., 2022; Ahuja & Khurana et al., 2021). Findings in this study are in accordance: many participants noted increased challenges in their relationships due to either spending more time together (i.e., cohabitating) or less time together (i.e., having to social distance apart). However, while findings in previous literature emphasize the negative impact of the pandemic on relationships and mental health, our study indicated more mixed results. Among those cohabitating, some found the increased time together strengthened the relationships between partners and/or family members. Additionally, use of technology has allowed for continued communication and affection between romantic partners. For those dating, some participants utilized dating applications to meet new people and start new relationships. For those already in relationships, many used online platforms such as Zoom and Skype to maintain connection and intimacy with their partner.

In combatting negative impacts of the pandemic, older, Black, same-gender-loving women used both internal and external factors to actively cope, in accordance with Bowleg et al.’s (2004) conceptual model of active coping among Black lesbians. Internal factors associated with active coping included using telehealth and online resources. Differing from previously published reports on telehealth experiences during the pandemic that found low satisfaction (Fisher et al., 2020), participants had largely positive encounters with telehealth visits for mental health. Telehealth eliminated some healthcare barriers experienced by older, Black, same-gender-loving women, leading some to even prefer telehealth. Along with telehealth, participants engaged in self-care skills and hobbies to promote active coping, many of which involved accessing online resources. Ability to practice such skills, however, was often dependent on the participants’ ability to work from home.

External factors associated with active coping included accessing mutual aid and social support. Since those in multiply marginalized populations are systematically underserved, they often depend on their own community to gain access to services and resources, with increased social support in aging marginalized communities producing better health outcomes (Van Vleet, 2021). While previous literature theorized that COVID-19 and social isolation may cut ties to LGBTQ networks (Philpot et al., 2021), our study indicates resilience among the Black LGBTQ community. Many participants engaged in food-related mutual aid as both providers and receivers. Social interaction and support were enhanced through online resources, allowing participants to maintain connections to larger community groups and spiritual centers. The combination of both internal and external factors promoted active coping among many of the participants in the study; most notably demonstrating the protective effects of online resources and access to social support on mental health. While most coping strategies indicated in the study were positive, many participants still noted feelings of isolation and desire for more community. Thus, utilizing technology and online resources should be prioritized to create and maintain support among Black, same-gender-loving women, promoting mental health outcomes especially during times of isolation.

This study is limited by its methodology as a cross-sectional study utilizing focus group discussions. Focus group discussions are susceptible to social desirability bias, which may have led individuals to provide responses that conform to societal norms or expectations, rather than reflecting their true beliefs or behaviors (Krueger & Casey, 2015). This may have compromised the accuracy of the findings. Additionally, because of the cross-sectional design, this study is limited by a lack of ability to explore and describe changes in experiences among participants over time (Wang & Cheng, 2020).

Despite these limitations, the study provides crucial data on the assets and unmet needs of an understudied group during the COVID-19 pandemic: older, Black, same-gender-loving women. The intersection of these social categories produces unique experiences and barriers, demonstrating the need for continued research into both the challenges faced by older, Black, same-gender-loving women, as well as their resilience capacity. Since the conclusion of data collection, a record number of anti-justice laws have been introduced (i.e., anti-LGBTQ, anti-Black, anti-Critical Race Theory, anti-women), increasing this population’s experiences with discrimination across all axes, magnifying existing barriers to care, and exacerbating the negative impacts presented in this study (Choi, 2023; Narea, 2022; Robinson, 2022). Therefore, it is now especially crucial to address knowledge gaps, adopt an intersectional lens, and provide more support for older, Black, same-gender-loving women to promote health equity in the future.

## 5. Conclusion

This study explored how older, Black, same-gender-loving women discussed their experiences during the COVID-19 pandemic. COVID-19 is an intersectional phenomenon; multiple structures of oppression intersect in the lives of older, Black, same-gender-loving women, shaping both determinants and consequences of the pandemic. Discrimination shaped experiences with healthcare outside of and during the pandemic, through various barriers and lack of access to providers of preferred social categories. The complex trauma experienced during the Trump Administration, COVID-19 lockdowns, and racial unrest after the murder of George Floyd had generally negative impacts across daily lives, relationships, and mental health. However, these were mitigated for some through positive coping strategies, including online resource use, mutual aid, and telehealth experiences. Interventions to mitigate future harms to this group should promote these existing assets by focusing on capacity building within communities. This includes scaling up online social supports, assisting mutual aid networks, providing safe ways to access healthy foods and exercise, and educating trusted community members to ameliorate distrust. Additionally, increasing the amount of female, Black, and/or LGBTQ providers is a crucial first step in improving healthcare outcomes and trust in this population. To promote health equity, future research should prioritize elucidating the healthcare and pandemic experiences among various multiply marginalized individuals. Specific to this demographic, future research should prioritize longitudinal studies which focus on older, Black, same-gender-loving women. To build upon these initial exploratory findings, in-depth interviews may be helpful for uncovering more nuance in these themes.

## Figures and Tables

**Table 1:** Demographic information for focus group participants (N = 102).

Measure	Category	N (%)
**Age**	Min	40
	Max	80
	Mean	57
**Race**	Black or African American	100 (98.03 %)
	Prefer not to answer	1 (0.98 %)
	Missing	1 (0.98 %)
**Sexual Orientation** ^a^	Lesbian	88 (86.27 %)
	Queer	12 (11.76 %)
	Same-gender-loving	10 (9.80 %)
	Gay	8 (7.84 %)
	Bisexual	3 (2.94 %)
	Another identity not listed	2 (1.96 %)
	Pansexual	1 (0.98 %)
**Gender** ^a^	Female or women	102 (100 %)
	Another Identity	4 (3.92 %)
	Gender nonbinary or genderqueer or genderfluid	3 (2.94 %)
**Education**	Master’s Degree	42 (41.17 %)
	Bachelor’s Degree	30 (29.41 %)
	Doctoral	10 (9.8 %)
	Some College	6 (5.88 %)
	Associates Degree	5 (4.9 %)
	Other	4 (3.92 %)
	Professional Degree (eg.MD, JD)	4 (3.92 %)
	Missing	1 (0.98 %)
**Employment**	Employed	45 (44.11 %)
	Retired	32 (31.37 %)
	Self-employed	13 (12.74 %)
	Unemployed	8 (7.84 %)
	Other	6 (5.88 %)
	Part-time employed	6 (5.88 %)
	Receiving unemployment	1 (0.98 %)
**Current Relationship Structure**	Single	47 (46.07%)
	Married	27 (26.47 %)
	Other	18 (17.64 %)
	Widowed	1 (0.98 %)
	Missing	9 (8.82 %)
**Household Composition**	Partners	44 (43.31 %)
	Children	21 (20.58 %)
	Friends	12 (11.76 %)
	Parents	11 (10.78 %)
	Other	10 (9.8 %)
	Siblings	10 (9.8 %)
	None of the above individuals live in my household	37 (36.27 %)

a Percentages do not sum to 100 % because participants were able to select more than one response.

**Table 2:** Themes and contributing concepts resulting from qualitative analysis of FGD data.

Theme	Contributing Concepts	Summary
**Determinants of Health**	**Intersectionality:** Participants note that their intersectional social categories significantly affected their experiences navigating healthcare and the pandemic.	Participants discuss the emergent properties of intersectionality in the context of the pandemic. Participants noted higher levels of stress due to the political and racial dynamics of 2020. Participants felt as though they were being more cautious than White counterparts regarding risk reduction.
	**Systematic Barriers:** Many participants experienced barriers to healthcare.	Many participants had negative experiences with healthcare due to discrimination with regard to race, gender, and/or sexual orientation. Participants noted the lack of resources accessible to communities of color as a significant barrier.
	**Providers:** Most participants indicated they had preferences regarding gender, race, sexuality and/or age when selecting a care provider.	The preference most indicated to be of most importance was the provider be female. The second most important preference was the provider be Black. The third most important preference was the provider be LGBTQ or LGBTQ-affirming. Age preferences varied among participants.
	**Medical and Governmental Distrust:** Many participants indicated some level of distrust due to historical and current discrimination.	Distrust informed opinions around the COVID-19 vaccine: most participants wanted to ‘wait-and-see’. Some participants would trust the vaccine once certain public figures take it.
**Impacts of the Pandemic**	**Health Impacts:** Impacts to mental health were more frequently mentioned than impacts to physical health.	Mental impacts were generally negative, due to increased stress and unpredictability. When physical impacts were mentioned, they were generally negative, due to less physical activity.
	**Impacts on Daily Life:** The most common impact of the pandemic was on the daily habits and behaviors of participants.	Participants indicated they were doing less or different forms of physical activity. Participants were overeating and snacking more. Participants’ hygiene habits were negatively affected by isolation. Participants were able to travel less.
	**Relationship Impacts:** The pandemic had pronounced effects on the relationships between the participant and others in their lives.	Those already in intimate relationships began spending more time together, which had varying effects. Those attempting to date found it harder after the pandemic. Ability to socialize with friends and family was thwarted.
	**Positive Impacts:** Despite more negative impacts mentioned, there were some instances of positive impacts of the pandemic.	Positive impacts were most related to more time and freedom due to the ability to work from home.
**Maintenance during the Pandemic**	**Coping:** More participants indicated positive coping skills than negative ones.	Positive coping strategies usually related to taking care of one’s mental health through self-care skills. Many participants indicated utilizing online resources to cope with the pandemic, such as Zoom, FaceTime, Pinterest, and YouTube. Negative coping strategies usually related to not taking care of one’s physical health.
	**Support:** Most support received during the pandemic was economic or resource related.	Mutual aid was the most common type of support received, followed by governmental aid.
	**Telehealth:** Telehealth was mainly accessed for mental health services and most experiences were positive.	Most use of telehealth was for mental health and characterized as positive, or even preferred over in-person. When characterized as negative, the health issue was most likely for physical health
